# Evolving our understanding of trained immunity

**DOI:** 10.7554/eLife.106029

**Published:** 2025-11-05

**Authors:** Eva Kaufmann, Yahya Sohrabi, Jorge Domínguez-Andrés, Boris Novakovic, Mihai G Netea, Jos WM van der Meer

**Affiliations:** 1 Meakins-Christie Laboratories, RI-MUHC, and Faculty of Dental Medicine and Oral Health Sciences, McGill University Montreal Canada; 2 Department of Biomedical and Molecular Sciences, Queen’s University Kingston Canada; 3 Department of Cardiology I – Coronary and Peripheral Vascular Disease, Heart Failure, University Hospital Münster Münster Germany; 4 Department of Internal Medicine and Radboud Center for Infectious Diseases, Radboud University Medical Center Nijmegen Netherlands; 5 Murdoch Children’s Research Institute and the Department of Paediatrics, University of Melbourne, Royal Children’s Hospital Parkville Australia; 6 Department of Immunology and Metabolism, Life and Medical Sciences Institute (LIMES), University of Bonn Bonn Germany

**Keywords:** innate immune cells, epigenetic changes, trained immunity, immunotherapy

## Abstract

The articles in this focus issue discuss progress towards a more complete understanding of memory in the innate immune system, and efforts to exploit “trained immunity” for the development of new vaccines and therapeutics.

Most scientific breakthroughs begin with an observation that initially defies explanation. One stunning example of this is the remarkable effectiveness of a vaccine called Bacillus Calmette-Guerin (BCG), which is still the only vaccine against tuberculosis. Since its introduction in 1921, the BCG vaccine has saved many more lives than can be attributed to protection against tuberculosis alone (de Castro et al., 2015). The mechanism behind this mystery remained elusive until 2012, almost a century later, when it was discovered that innate immune cells could be "trained" by BCG vaccination to respond more robustly to a variety of threats (Kleinnijenhuis et al., 2012). For decades, it had been known that adaptive immune cells have a memory function, but it was assumed that innate immune cells did not.

Adaptive immunity relies on specialized T and B cells that, upon recognizing specific antigens, generate both immediate defenders and long-lived memory cells. These memory cells enable rapid and robust responses to subsequent encounters with the same pathogen. This sophisticated defense system emerged relatively recently in evolutionary terms, first appearing in jawed fish around 500 million years ago. The development of the innate immune system, on the other hand, can be traced back further, to 1 billion years ago, when the earliest complex life forms first appeared on Earth (Martins et al., 2023). It may even be argued that a rudimentary form of innate immunity is present in bacteria, which build an anti-viral response against bacteriophages.

While the possibility that mammals possess a form of immune memory within their innate immune system was first proposed in the 1960s (Mackaness, 1962; Mackaness, 1964), this description was largely overshadowed by the rapid advances in the knowledge of adaptive immunity at that time – a field that has since been honored by numerous prizes (including the Nobel Prizes for Physiology and Medicine in 1960, 1972, 1984, 1987, 1996, and 2025). Interest in the concept of innate immune memory faded until the 2000s, when epigenetically mediated LPS-induced tolerance (Foster et al., 2007) and BCG-induced training (Kleinnijenhuis et al., 2012) in myeloid cells was discovered.

The reviews and research articles in this focus issue highlight the remarkable progress that has been made in our understanding of trained immunity, and also identify critical gaps in our knowledge of the concept.

In contrast to the adaptive immune system, where memory relies on the presence of specialized cells, memory in the innate immune system is due to alterations in the antimicrobial and proinflammatory capacities of cells, driven by metabolic (Cheng et al., 2014) and epigenetic (Saeed et al., 2014) reprogramming. The fact that trained immunity does not rely on specialized cells means that virtually any cell can learn and adapt, underscoring the idea that "every cell is an immune cell”. Recent research has revealed the conservation of this memory in long-lived structural (Naik et al., 2017) and hematopoietic (stem) cells (Kaufmann et al., 2018; Mitroulis et al., 2018; Yao et al., 2018). These and many other advances have expanded our understanding of both fundamental pathological processes in numerous diseases, as detailed in this focus issue, and potential new therapies. The first clinical applications are currently emerging.

Evidence of trained immunity has expanded beyond BCG to include agents like β-glucan, lipopolysaccharide (LPS), heme, and various ligands and microbes, which have shown promise in both laboratory models and clinical settings (reviewed in detail by Domínguez-Andrés et al., 2023). Applications range from non-specific vaccine benefits to cancer therapies (Jurado et al., 2025).

Defining characteristics of trained immunity are the persistence of its epigenetic changes after the initial stimulus has ceased and a return to baseline cellular activation status – hallmarks that distinguish it from priming or differentiation (Divangahi et al., 2021). This flexibility in innate immune cells may confer advantages over adaptive immunity, especially in short-lived or vulnerable organisms like plants and invertebrates. While adaptive memory is stable and long-lasting, trained immunity may be more dynamic and capable of responding quickly to environmental changes.

## Challenges and open questions

Over the past decade, a series of breakthroughs has revealed new questions and challenges that kept the field moving forward. How long does trained immunity last, and does the longevity of the memory response depend on the type of receptors engaged? Can innate memory be re-reprogrammed after initial training? Is there an evolutionary link between trained immunity and pathogen adaptation? Answers to these questions are needed to help researchers design new vaccines and therapies.

Research on plants and invertebrates has quietly, yet significantly, contributed to our understanding of innate immune memory (Kachroo and Robin, 2013; Kurtz and Franz, 2003), uncovering important mechanisms of immune adaptation that warrant further investigation in mammals. For example, insights from studies of cytokine signaling and vertically transmitted immunity may help to improve our understanding of mammalian immunology.

There are other unanswered questions. Was trained immunity an evolutionary adaptation in organisms lacking adaptive immunity? It may have originated as an epigenetic "scar" from past infections, providing a survival advantage. Could pathogens themselves evolve epigenetic strategies to bypass host defenses? These evolutionary questions remain largely unexplored and may hold the key to understanding the origins of immune memory.

The distinction between host-beneficial trained immunity and maladaptive innate immune memory (Khan et al., 2020; Li et al., 2022) requires detailed exploration. While trained immunity enhances immune responses, maladaptive memory can result in excessive inflammation or weakened defenses against pathogens. Understanding this balance is critical for developing safe and effective treatments.

Epigenetically conserved memory mechanisms can be triggered by various stimuli, including infections, vaccines and inflammatory mediators. In contrast to laboratory conditions, humans are constantly exposed to diverse environmental stimuli, which adds complexity to the study of trained immunity in real-world scenarios. Understanding how multiple exposures interact will, therefore, be vital for future therapeutic designs.

Another important question is: can trained immunity be enhanced or erased? Studies show that trained immunity can last for over a year in mice (Khan et al., 2020), which is a significant fraction of their lifespan, and for at least multiple months in humans (Kleinnijenhuis et al., 2012), but specific endpoint-defining studies are still missing. It remains unknown if innate memory can persist for a lifetime and, if so, under what conditions. This information is crucial for designing long-lasting vaccines and therapies that do not compromise immune flexibility.

The potential of trained immunity extends beyond infectious diseases. It offers hope for treating conditions where adaptive immune strategies have failed, such as some cancers, autoimmune diseases, and chronic inflammatory disorders. Emerging technologies and targeted delivery systems (Schrijver et al., 2023) will soon transform how trained immunity is harnessed in the clinic, enabling therapies to become more precise and effective.

Trained immunity represents an ancient, yet revolutionary concept in immunology. It challenges long-held beliefs and opens exciting avenues for understanding and treating diseases where traditional adaptive immune strategies were not the ultimate answer. Improving our knowledge of the mechanisms that underpin innate immune memory and applying it to new therapies and vaccines demands sustained collaboration and innovation.

Together, we can lead the next breakthrough in medicine.
